# The Anti-Constipation Effects of Raffino-Oligosaccharide on Gut Function in Mice Using Neurotransmitter Analyses, 16S rRNA Sequencing and Targeted Screening

**DOI:** 10.3390/molecules27072235

**Published:** 2022-03-30

**Authors:** Yuxuan Liang, Yu Wang, Peng Wen, Yongchun Chen, Dongmei Ouyang, Da Wang, Bin Zhang, Jie Deng, Yanhong Chen, Yuanming Sun, Hong Wang

**Affiliations:** 1 College of Food Science, South China Agricultural University, Guangzhou 510642, China; l402972616@163.com (Y.L.); pwen@scau.edu.cn (P.W.); 20172123002@stu.scau.edu.cn (Y.C.); ouydm2019@163.com (D.O.); 20172013009@stu.scau.edu.cn (D.W.); zhangbin2018@stu.scau.edu.cn (B.Z.); dengjie@stu.scau.edu.cn (J.D.); 2 Guangdong Provincial Key Laboratory of Food Quality and Safety, Guangzhou 510642, China; 3 Guangzhou Institute for Food Inspection, Guangzhou 511400, China; xxwangyu@163.com (Y.W.); chenyanhom@outlook.com (Y.C.); 4 Guangdong Laboratory for Lingnan Modern Agriculture, Guangzhou 510642, China

**Keywords:** raffino-oligosaccharide, constipation, gut microbiota, targeted screening, network pharmacology, molecular docking

## Abstract

Raffino-oligosaccharide (ROS), the smallest oligosaccharide of the raffinose family, is a novel food ingredient. However, the anti-constipation effects of ROS remain obscure. This study investigates the anti-constipation effects of ROS based on the loperamide-induced mice model and reveals the underlying mechanism using constipation parameters, neurotransmitter level, 16S rRNA sequencing, and the targeted screening strategy. The prevention effects were firstly investigated by the gastro-intestinal transit rate experiment (50 mice) and defecation status experiment (50 mice), which were divided into five groups (*n* = 10/group): blank, model, and low-, medium- and high-dose ROS. Furthermore, the slow-transit constipation experiment (blank, model, and high-dose ROS, *n* = 10/group) was conducted to illustrate the underlying mechanism. The results showed that ROS aided in preventing the occurrence of constipation by improving the gastro-intestinal transit rate and the defecation frequency in mice, and ROS significantly reduced the serum levels of vasoactive intestinal peptide (VIP). In addition, ROS regulated the diversity and structure of intestinal flora. Among them, one specific family and six specific genera were significantly regulated in constipated mice. The targeted screening revealed that 29 targets related to the anti-constipation effects of ROS, indicating ROS may play a role by regulating multiple targets. Furthermore, the network pharmacology analysis showed that Akt1, Stat3, Mapk8, Hsp90aa1, Cat, Alb, Icam1, Sod2, and Gsk3b can be regarded as the core anti-constipation targets. In conclusion, ROS could effectively relieve constipation, possibly by inhibiting the level of neurotransmitters and regulating the gut flora in mice. This study also provides a novel network pharmacology-based targeted screening strategy to reveal the anti-constipation effects of ROS.

## 1. Introduction

Constipation is a complex gastrointestinal syndrome, the primary features of which include infrequent bowel movements, prolonged gastrointestinal transit time, and difficulty with defecation, involving the regulation of multiple targets [1,2,3,4,5]. Extensive studies reported that the alteration in the intestinal flora is associated with the symptoms of constipation such as slow gastrointestinal transit and abnormal defecation [6,7,8]. At present, the use of oligosaccharides as a dietary supplement to alleviate constipation has received increased attention from researchers [9,10]. Some oligosaccharides (fructo-oligosaccharide, galacto-oligosaccharide, isomalto-oligosaccharide, and Konjac mannan oligosaccharides) have been reported to possess good effects for constipation relief. For instance, they significantly improved gastrointestinal transit and defecation by the regulation of gut microbiota and enteric nervous system in constipated mice or rats [9,11,12].

Raffino-oligosaccharide (ROS) is an α-1,6-linked galactosides trisaccharide (consisting of D-galactose, D-glucose, and D-fructose) [10]. It is difficult to digest completely in mammals, and the indigestible portion of ROS can be utilized by gut flora [13,14]. A previous study indicated that the average percentage of the administrated ROS collected in the terminal ileum in humans was 97% [15], indicating the majority of ROS provides a basis for the intestinal flora of the large intestine to use ROS as a substrate. However, Watanabe used the caecectomy and administration of antibiotics to study the health-promoting effect ROS, which indicted a postabsorptive mechanism that is independent of the utilized by the gut microbiota may participate in the effect of ROS [16].

With the development of bioinformatics, many target data associated with compounds and diseases have been systematically collected by some databases. Among them, PubChem collects information on more than 100 million compounds, including reports on the biological activity of ROS. DrugBank provides up-to-date information on constipation drugs and related targets. On this basis, the targeted screening becomes a feasible research strategy, which is a new bioinformatics method based on the matching of ROS’s targets and constipation’s targets. The acquisition of intersecting targets helps to further understand molecular mechanisms and provides a systematic perspective. As more data are collected in the database, the more comprehensive the targeted screening can reveal the relevant mechanisms. Based on the above literature, we hypothesized that ROS may play an anti-constipation role by regulating gut microbiota, enteric nervous system, and constipation-related target.

In this study, the anti-constipation role of ROS was firstly investigated by evaluating the gastro-intestinal transit rate (GI) and defecation status in mice. Then, the regulation characteristics of ROS were clarified by the level of neurotransmitters and 16S rRNA sequencing. Lastly, the targeted screening strategy was further used to illustrate the gut prebiotic properties of ROS.

## 2. Results

### 2.1. Effects of ROS on the Gastro-Intestinal Transit Rate and Defecation

**Figure 1:**
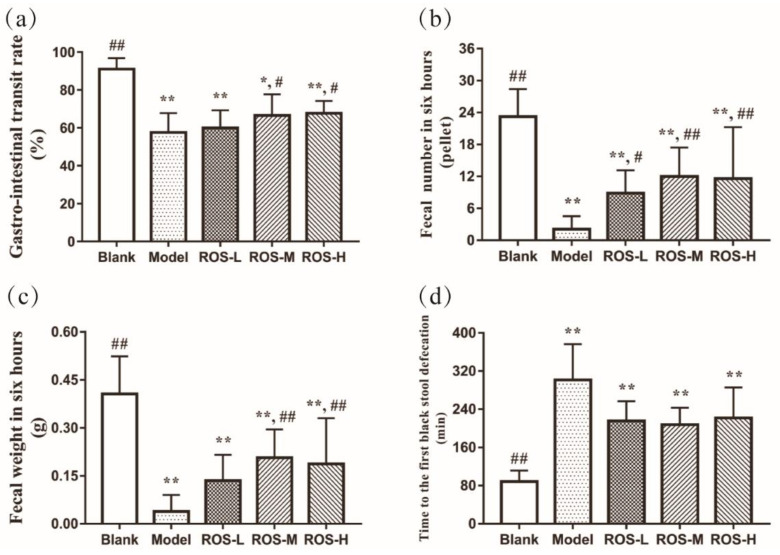
Gastro-intestinal transit rate (**a**) and defecation status: the weight (**c**) and number (**b**) of defecation events in six hours and the time to the first black stool defecation (**d**) of mice. Blank: distilled water treated; Model: Loperamide treated; ROS-L: low-dose ROS treated; ROS-M: medium-dose ROS treated; ROS-H: high-dose ROS treated. * *p* < 0.01 than in the blank group; ** *p* < 0.01 than in the blank group; # *p* < 0.05 and ## *p* < 0.01 than in the model group.

Figure 1 showed the GI and defecation status in the assayed mice. The model group exhibited slower parameters (than in the blank group, *p* < 0.05), indicating that the constipated model was built. The GI was not significantly increased in the low-dose ROS group than in that of the model group, while the GI was increased in the medium-dose and high-dose ROS groups (Figure 1a, *p* < 0.05). Although the ROS groups did not significantly reduce the time to the first black stool defecation (Tfbsd) compared with the model group, low-ROS, medium- ROS, and high-ROS shortened the time to Tfbsd by 86.0 min, 93.7 min, and 79.6 min, respectively (compared to the model group) (Figure 1d). Notably, the weight of defecation in the feces (Fw6h) and the number of defecation events in six hours (Fn6h) were significantly improved in all ROS groups than that in the model group (Figure 1b,c, *p* < 0.05). In a word, the results of GI and defecation revealed that ROS could effectively accelerate the intestinal peristalsis and defecation status to prevent the occurrence of constipation at the dose set in the experiment.

### 2.2. Effects of ROS on Serum Neurotransmitter

**Figure 2:**
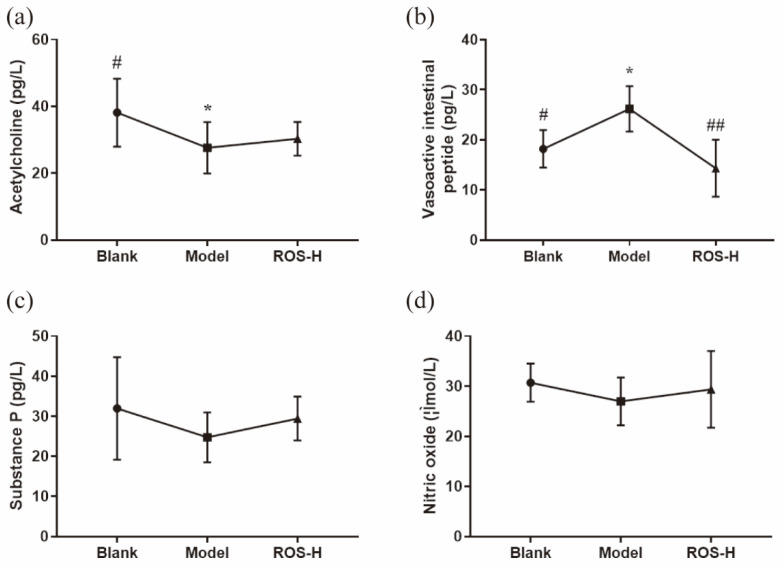
Effects of ROS on serum level of acetylcholine (**a**), vasoactive intestinal peptide (**b**), substance P (**c**), and nitric oxide (**d**) in mice. Blank: distilled water treated; Model: Loperamide treated; ROS-L: low-dose ROS treated; ROS-M: medium-dose ROS treated; ROS-H: high-dose ROS treated. * *p* < 0.01 than in the blank group; # *p* < 0.05 and ## *p* < 0.01 than in the model group.

The effects of ROS on relieving constipation were further evaluated by measuring serum neurotransmitters (acetylcholine (Ach), substance P (SP), nitric oxide (NO) and vasoactive intestinal peptide (VIP)) in mice. The results showed that no significant difference was observed in the level of SP and NO among all groups (Figure 2c,d). Although the levels of Ach exhibited a significant decrease in the model group (compared to the blank group, *p* < 0.05) (Figure 2a), it was not significantly increased in the ROS group (compared to the blank group). Interestingly, the levels of VIP were significantly increased in the model group (*p* < 0.05) compared with the blank group, while this trend was reversed after the administration of ROS (Figure 2b, *p* < 0.05).

### 2.3. Effect of ROS on the Diversity of the Rectal Microbiota

**Figure 3:**
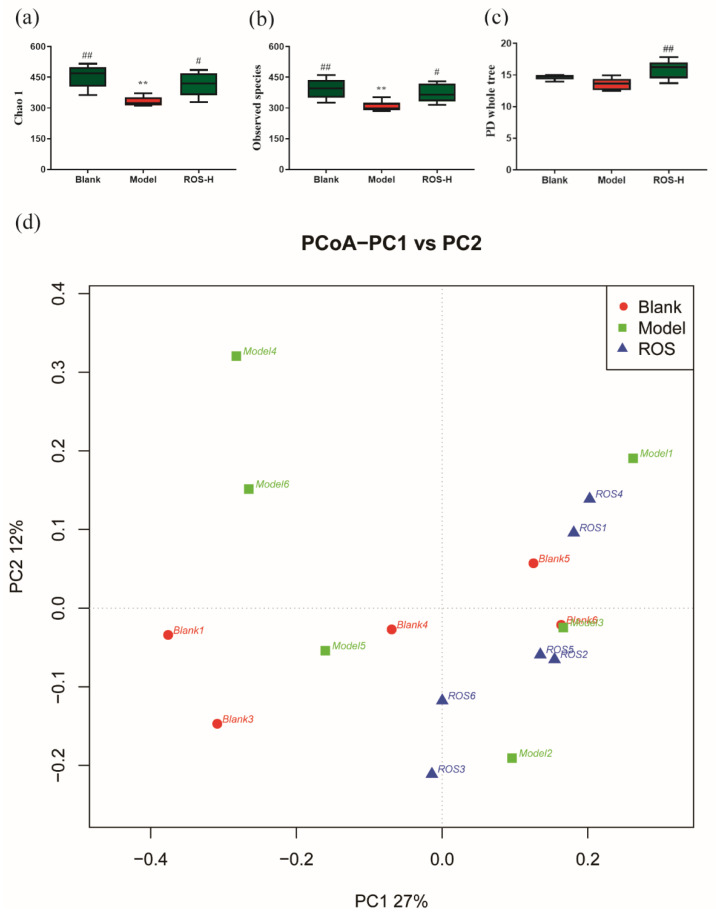
Alpha diversity indices (**a**–**c**); blank-distilled water treated; model—Loperamide treated; ROS-H: high-dose ROS treated. Beta diversity index (**d**); ROS1-6: high-dose ROS treated. ** *p* < 0.01 than in the blank group; # *p* < 0.05 and ## *p* < 0.01 than in the model group.

To evaluate the role of ROS on the composition of gut microbiota, metagenomic alpha diversity was performed using 16S rRNA sequencing. Compared with the model group, the blank group showed statistical differences in the species richness (Figure 3a–c), which indicated that the species richness of the intestinal flora was decreased when constipation was induced by loperamide (*p* < 0.05). After ROS treatment, the Chao-1, PD whole tree, and observed species indices were remarkably improved (compared to the model group, *p* < 0.05), while no statistical differences were observed between the blank group and ROS group, indicating the species richness of intestinal flora recovered after ROS administration. Beta diversity (PCoA) was also evaluated using the unweighted uniFrac matrices. Overall differences were observed in microbial compositions among different groups (Figure 3d). These results revealed that the ROS administration could change the structure of the gut flora in constipated mice.

### 2.4. Effect of ROS on the Level of the Rectal Microbiota

**Figure 4:**
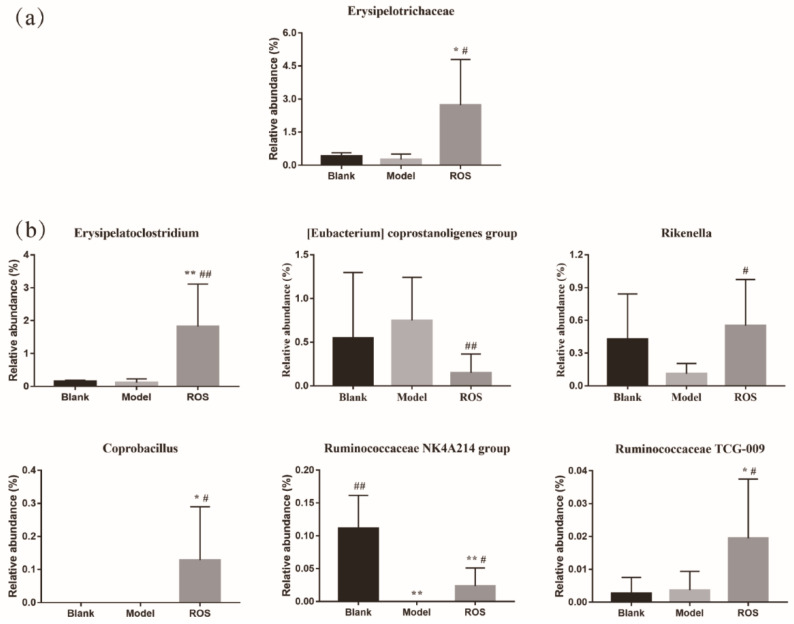
Alteration in the level of family (**a**) and alteration in the level of genus (**b**). Blank: distilled water treated; Model: Loperamide treated; ROS: high-dose ROS treated. * *p* < 0.05 and ** *p* < 0.01 than in the blank group; # *p* < 0.05 and ## *p* < 0.01 than in the model group.

At the family level, analysis of variance showed the level of Erysipelotrichaceae was increased in the ROS group compared with that of the model group (Figure 4a, *p* < 0.05). At the genus level, ROS administration significantly elevated the levels of *Erysipelatoclostridium*, *Rikenella*, *Coprobacillus*, *Ruminococcaceae NK4A214 group*, and *Ruminococcaceae TCG-009* than in that of the model group, while ROS administration significantly reduced the level of *(Eubacterium) coprostanoligenes group* in comparison with that of the model group (Figure 4b, *p* < 0.05).

### 2.5. Targeted Screening and Analysis of Protein–Protein Interaction Network

**Figure 5:**
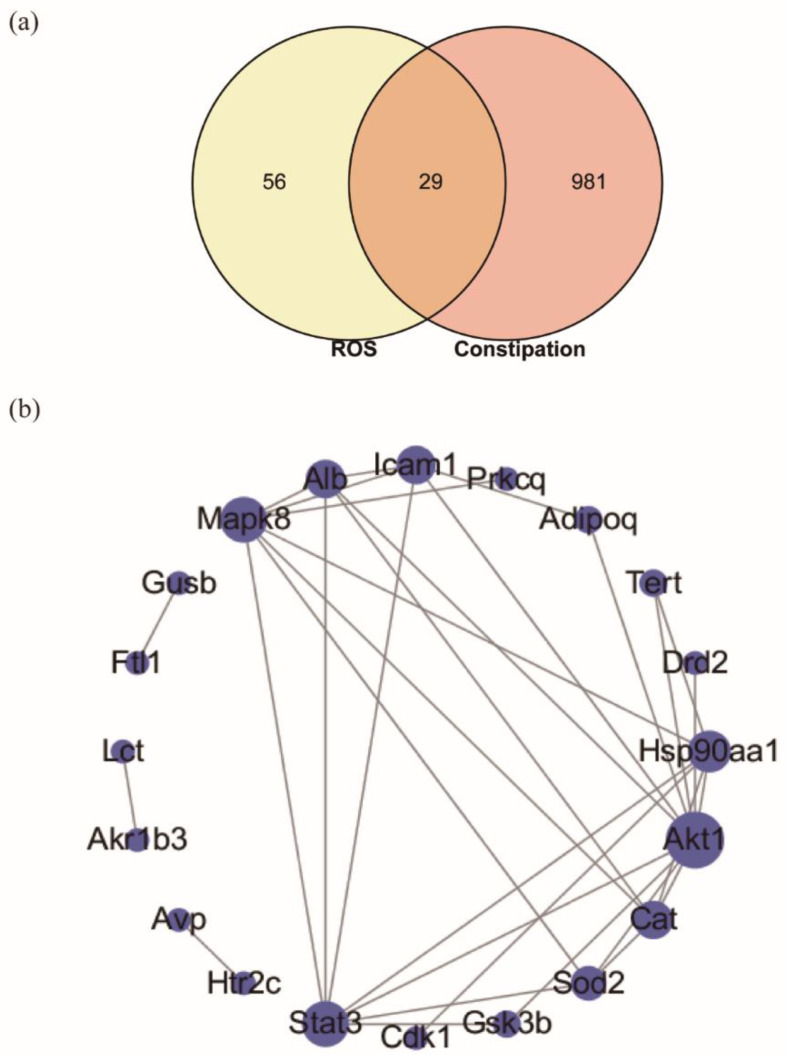
(**a**) Intersection of ROS candidate targets and constipation targets, and (**b**) PPI network visualization of overlapped targets, and the size of nodes represents the value of the degree.

Through the targeted screening, 85 candidate targets (*Mus musculus*) of ROS and 1143 therapeutic targets related to constipation were screened. The intersection of ROS targets and constipation-related targets are shown with a Venn diagram (Figure 5a), and the detailed information for overlapped targets is shown in Table S1. The result showed that the intersection of ROS and constipation contains 29 overlapped targets. Then, the protein–protein interaction (PPI) network affected by 29 overlapped targets was constructed (Figure 5b and Table S2), which consists of 29 nodes and 32 edges. Moreover, the PPI network analysis was performed, and the network parameters for degree was shown in Table 1. The network analysis revealed that the average degree value for overlapped targets was 3.2. There were eight targets more than the average degree values, including Akt1, Stat3, Mapk8, Hsp90aa1, Cat, Alb, Icam1, and Sod2. Therefore, these eight targets were regarded as the core targets of ROS for anti-constipation in the PPI network.

### 2.6. Analysis for Functional Enrichment and ROS-Target-Pathway Network

**Figure 6:**
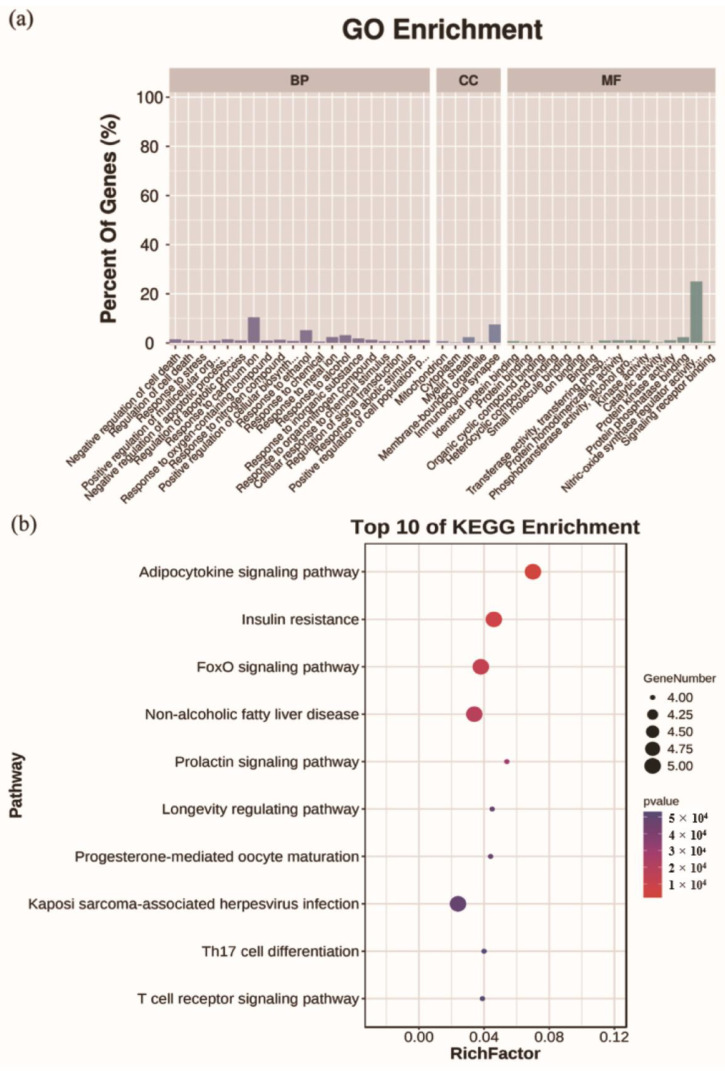
GO terms and KEGG pathways enrichment analysis of the overlapped targets. (**a**) Gene Ontology (GO) terms for biological processes (BP), cellular components (CC), and molecular function (MF), (**b**) KEGG pathways.

**Figure 7:**
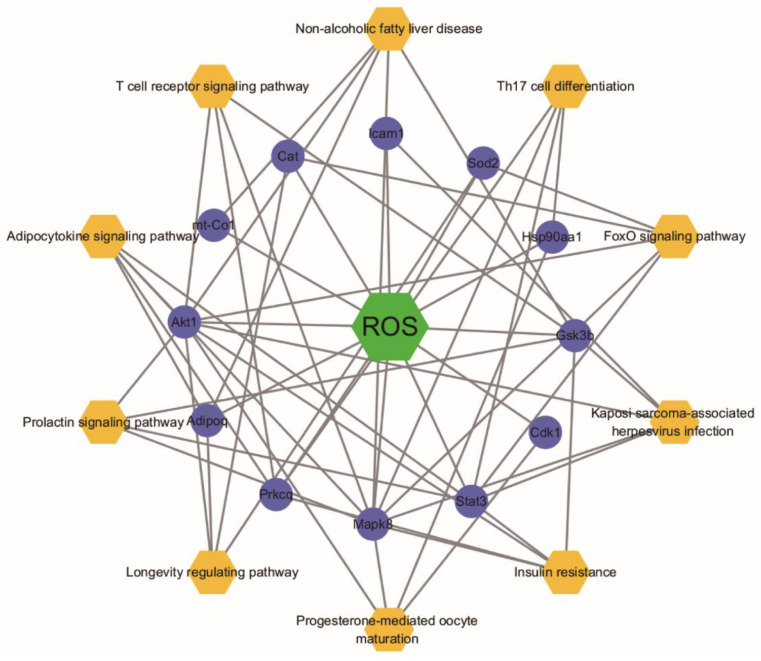
ROS-Target-Pathway network. Orange hexagons represent pathways, the green square represents ROS and purple rounds represent targets.

Based on the results of targeted screening, the functional enrichment analysis was performed. The results showed that 371 Gene Ontology (GO) terms were identified (Tables S3–S5), and the top 20 biological processes (BP), 5 cellular components (CC), and 16 molecular function (MF) were selected based on the *p*-value and percentage of enriched genes (Figure 6a). A total of 29 overlapped targets were enriched in the BP, mainly including the regulation of cell death and response to stress. The results indicated that overlapped targets were distributed in different CC, such as mitochondrion and cytoplasm. MF was mainly reflected in the binding of protein, drug, and organic cyclic compound. KEGG pathway enrichment analysis showed that 66 signal pathways were enriched (Table S6). Of them, the top 10 KEGG pathways were selected (Figure 6b), including adipocytokine signaling pathway, insulin resistance, foxO signaling pathway, non-alcoholic fatty liver disease and prolactin signaling pathway. Furthermore, the selected top 10 pathways were used to construct the ROS-target-pathway (R-T-P) network (Figure 7), revealing the average degree values of targets were 3.7 (Table S7). Among them, Akt1 (degree = 9), Mapk8 (degree = 9), and Stat3 (degree = 6) showed higher degree values, indicating more central nodes in the R-T-P network. Therefore, these targets can be regarded as the core targets of ROS for anti-constipation.

### 2.7. Homology Modeling and Molecular Docking

**Figure 8:**
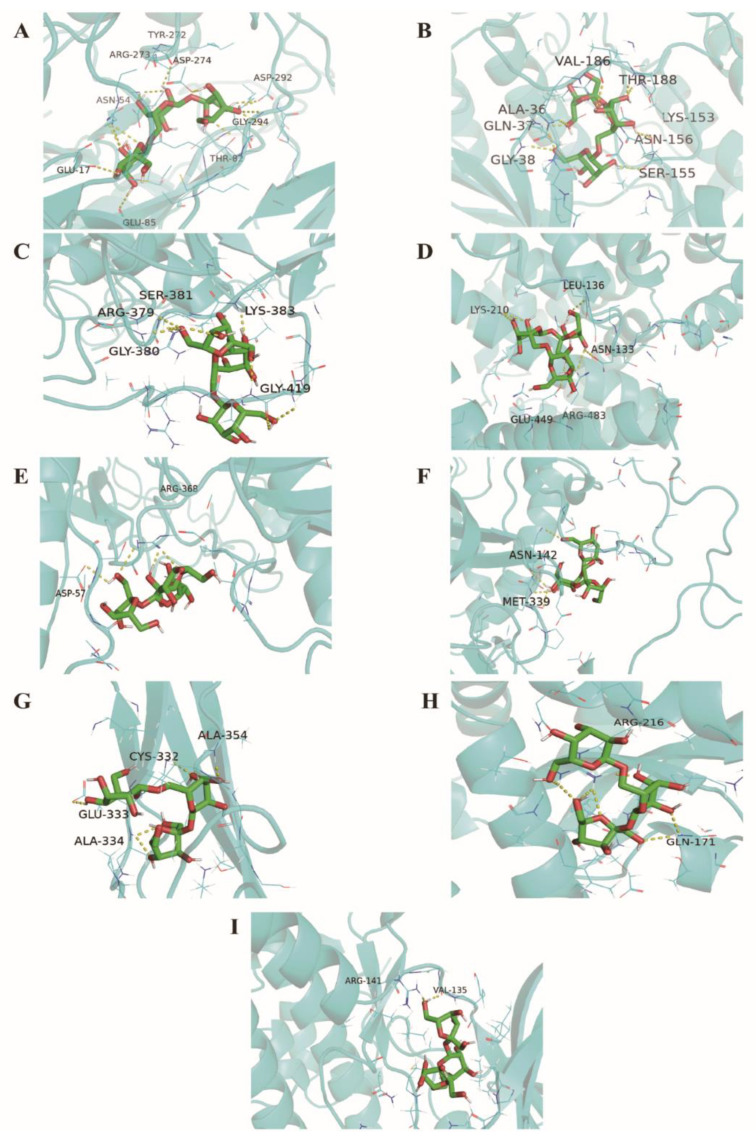
Binding pattern of ROS to Akt1 (**A**), Mapk8 (**B**), Stat3 (**C**), Alb (**D**), Hsp90aa1 (**E**), Cat (**F**), Icam1 (**G**), Sod2 (**H**), and Gsk3b (**I**).

To verify the results of the targeted screening, the core targets (Akt1, Stat3, Mapk8, Hsp90aa1, Cat, Alb, Icam1, Sod, and Gsk3b) were selected from the PPI network and R-T-P network for further molecular docking. The crystal structure for Stat3 was available from the Protein Data Bank (PDB entry: 4E68), and the crystal structure for Gsk3b was also obtained from the Protein Data Bank (PDB entry: 6AE3). As the crystal structures of some target proteins were not found, homology modeling was carried out by Swiss-model (Akt1, Mapk8, Hsp90aa1, Cat, Alb, Icam1, and Sod2). Sequences for those proteins were acquired from the UniProt database. The results of homology modeling were shown in Table S8. The backbone structure of the protein was evaluated in the Ramachandran plot (Figure S1). The results of binding were shown in and Figure 8 and Table 1. We found that ROS showed a favorable binding affinity for the eight core targets above (the binding energy < −5 kJ/mol). Among them, Akt1, Mapk8, and Stat3 showed a stronger combination with ROS.

## 3. Discussion

The supplement of prebiotics and probiotics has been reported to be a practical method to influence intestinal function [9,17], for instance tagatose, inulin, *Bifidobacterium*, and *lactobacillus* [18,19,20]. The administrated ROS collected in the terminal ileum was 97%, the feature of which suggested that it possesses a potential prebiotic effect [13,14,15]. In this study, we found ROS shortened the time to the first black stool defecation, and it significantly improved the gastrointestinal transit rate and the defecation of six hours in constipated mice. We further investigated the underlying mechanism of ROS on constipation by gut microbiota, neurotransmitters, and targeted screening.

Constipation is a complex disease driven by complex interactions between multiple molecules. Lifestyle changes, such as dietary supplement (prebiotics and probiotics) and exercise status (mild to moderate intensity), are the key measures of prevention [19,20,21,22]. The primary symptoms of constipation are difficulty with defecation and a reduced frequency of defecation [23]. Therefore, we used key indices of constipation (GI, Tfbsd, Fw6h, and Fn6h) to assess the preventive effect of ROS on constipation. In the GI experiment, the medium-dose ROS and high-dose ROS effectively improved the GI in mice (Figure 1a, *p* < 0.05). In addition, the movement of gastrointestinal contents is detected by measuring the status of defecation [1]. In the defecation experiment, supplementation of ROS significantly regulated Fw6h and Fn6h in constipated mice (Figure 1b,c). For the Tfbsd, a reduced trend was observed in the all-ROS group compared with the model groups (Figure 1d).

The levels of neurotransmitters are closely related to the motility of the gastrointestinal tract [23,24]. The excitability neurotransmitters (SP and Ach) and inhibitory neurotransmitters (VIP and NO) were determined. The results showed that ROS effectively down-regulated the serum level of VIP (Figure 2b). VIP is an inhibitory neurotransmitter that relaxes gastrointestinal tract smooth muscles and thus restrains gastrointestinal motility [25]. These results revealed that ROS could reduce the level of VIP and improve the frequency and status of defecation in constipated mice.

At present, numerous studies have shown that microbiomes influence host health and behavior via multiple mechanisms [26,27]. Thus, to evaluate the changes of gut microbiota, the 16S rRNA of intestinal flora was implemented to explain the anti-constipation mechanism of ROS. The study revealed that ROS could significantly elevate the alpha diversity of the intestinal flora and could effectively alter the rectal microbiota structure (Figure 3a–c). Erysipelotrichaceae is known as being a butyrate-producing bacteria and butyrate was reported that could accelerate GI transit and maintain barrier integrity [28,29]. Our study revealed that ROS administration significantly promoted the relative abundance of Erysipelotrichaceae (Figure 4a). At the genus level, a previous study reported that *Erysipelatoclostridium* was relatively more abundant in constipated patients [30]. Paradoxically, the level of *Erysipelatoclostridium* in the constipated mice decreased significantly [31]. In this study, *Erysipelatoclostridium* was significantly increased after ROS treatment in constipated mice (Figure 4b). The increase in *Rikenella* in the intestine is related to the improvement of constipation [32], and *Coprobacillus* should help with bowel health and transit time and constipation [33]. In the study, these two genera were significantly increased by ROS administration (Figure 4b). Additionally, Ruminococcaceae is also known as being a butyrate-producing bacteria, and the feces of constipated people are featured with restrained Ruminococcaceae [29]. The reduction in Ruminococcaceae could decrease the validity of butyrate, thereby increasing the epithelial permeability, which is related to the regulation function of the immune cells and nerve cells in the gut [34]. In this study, we found that two genera (*NK4A214 group* and *TCG-009*) belonging to Ruminococcaceae were significantly increased after ROS administration (Figure 4b). We speculate that these two genera may have similar effects. Overall, ROS significantly regulated one family and six genera in constipated mice, suggesting that ROS may relieve constipation by regulating these bacteria.

To date, studies mainly focused on the action of oligosaccharides on the intestinal flora and related neurotransmitters [19,23,31]. However, focusing only on these aspects may result in missing out on key information. In this study, the targeted screening strategy was first adopted to reveal complementally the anti-constipation effects of ROS. It provides insight of bioinformatics and systems biology [35,36]. The targeted screening strategy is to map potential ROS targets to known constipation-related targets, thus obtaining the overlapped targets of ROS and constipation, which will help to discover the ‘relation’ or ‘effect’ between ROS and constipation. The results showed that 29 potential targets affected by ROS related to constipation (Figure 5a). Furthermore, the functional enrichment analysis (Figure 6a) revealed that BP gathered from regulation of cell death and response to stress, and CC mainly included mitochondrion and cytoplasm. MF was mainly enriched in the binding of protein, drug, and organic cyclic compound. As there is no related pathway named after constipation at present, we displayed the top 10 KEGG pathways affected by the overlapped targets (Figure 6b).

To further discover the key targets of constipation regulated by ROS, the network topological analysis was performed. The PPI network analysis showed that Akt1, Stat3, Mapk8, Hsp90aa1, Cat, Alb, Icam1, and Sod2 were regarded as the core targets of ROS for anti-constipation due to their higher degree value in the network (Figure 5b and Table 1). Moreover, the R-T-P network also contains these eight targets (Figure 7 and Table S7). The previous studies reported that these eight potential targets affected by ROS mainly involved the regulation of the inflammatory response, the immune process, and antioxidation [18,37,38,39]. Accumulating evidence has reported a strong interaction between the intestinal flora and immune [40]. A tight intestinal barrier helps to prevent the invasion of pathogenic elements, while the impaired intestinal barrier may contribute to gut inflammation [18,41]. Molecular docking technology was further used to verify the connection between ROS and core targets. The results showed that ROS had good binding and interaction with these core targets (Figure 8 and Table 1). In a word, ROS relieves constipation possibly by regulating the gut microbiota, reducing the inhibitory neurotransmitters, and regulating targets related to inflammation, immunity, and antioxidation.

## 4. Materials and Methods

### 4.1. Materials

ROS was acquired from the Beijing China Leader Biological Science and Technology Co. Ltd. (Beijing, China), and ROS included 94% raffinose. The information on the kit and loperamide is consistent with our previous study [19]. Loperamide and activated carbon meal solutions were prepared following the method described by Wang [1].

### 4.2. Arrangement of Animal Experiments

There are 130 male Kunming mice (20 ± 2 g) that were obtained from the Guangdong Medical Laboratory Animal Center (Guangzhou, China). The mice were maintained under temperature 25 ± 2 °C, humidity 50 ± 5%, 12 h light–dark cycle, and the standard mouse food (64% carbohydrate, 19% protein, and 17% fat) and water were provided to feed mice. The Ethics Committee of South China Agricultural University (SYXK2014-0136) approved all the protocols, grant number 2017009.

#### 4.2.1. Dosage Regimen

The low-, medium-, and high-dose settings of ROS (0.44, 0.89 and 1.77 g/kg·bw) for mice were equivalent to 5, 10, and 20 times of the maximum recommended dose for humans (5 g/60 kg·bw) by regulations of China Food and Drug Administration.

#### 4.2.2. GI Experiment

Fifty mice were randomly separated into five groups (*n* = 10); after adaptive feeding for seven days, each of the five mice was placed in a separate cage. The specific experimental workflow for this assay was followed. The blank and model group were given distilled water for seven days (gavage, once a day); the ROS groups were given different concentrations of ROS in the same manner, individually. The GI of each mouse was measured using previously described methods when the experiment ended [42,43].

#### 4.2.3. Defecation Experiment

Fifty mice were randomly divided into five groups after adaptive feeding for seven days. The specific experimental workflow for this experiment was the same as that described for the gastro-intestinal transit experiment. Loperamide (10 mg/kg) suspended in distilled water was prepared to induce constipation. Each mouse was maintained in a single cage at the beginning of index measurement. At the end of the experiment, Tfbsd, Fn6h, and Fw6h collected during the six hours was measured for each mouse [44].

#### 4.2.4. Arrangement of Slow-Transit Constipation Experiment

Thirty mice were randomly divided into three groups after adaptive feeding for seven days. A slightly modified version of the method was adopted to establish the slow-transit constipation model [45], and the ROS group received high dose-ROS. The distal rectum of the mice was collected, respectively, which contained formed feces.

### 4.3. 16S rDNA Sequencing

The paired-end sequenced (2 × 250) was performed on the HiSeq 2500 platform for 16S rRNA sequencing. The detailed information of the sequencing procedures was shown in the previous study [19].

### 4.4. Determination of Neurotransmitters in Serum

The contents of acetylcholine Ach, SP and VIP in the serum were measured using ELISA kits, and the level of NO in the serum was determined by the nitrate reductase method.

### 4.5. Collection of Candidate Target and Construction of the Network

For candidate target of ROS, the databases for TCMSP [46], Swiss Target Prediction [47], SymMap [48], and PubChem were used. For the therapeutic target of constipation, the targets were collected from the DrugBank 5.1.6 [49], GeneCards 4.14 [50], and DisGeNET 7.0 [51]. For the setting of target conditions, the gene correlation screening of the score of the GeneCards database was set to ≥3.328 (average score). The screening criterion of the score of the DisGeNET database ≥ 0.1. Furthermore, the STRING database was used to obtain a PPI network of intersection targets, and species of “Mus musculus” with a score ≥ 0.7 were selected.

### 4.6. Enrichment Analysis and Cytoscape Network Construction

Based on the overlapped targets, GO terms and KEGG pathway enrichment analysis were performed by using STRING bioinformatics resources [52]. Cytoscape 3.6 [53] was used to construct the PPI network and R-T-P network, and the Network Analyzer tool of Cytoscape was used to calculate the topological characteristics of the PPI network and R-T-P network, and the important indicator ‘Degree’ was selected for further analysis.

### 4.7. Preparation of Protein Structure

The core targets were screened under the condition that the ‘Degree’ of the node in the PPI network and R-T-P network was greater than the mean values. The protein sequences of the mouse (Akt1, Mapk8, Hsp90aa1, Cat, Alb, Icam1, and Sod2) were collected from the UniProt. As no crystal structures were available for them, SWISS-MODEL was used to construct the homology models for them [54,55,56]. The 3D structure of Stat3 and Gsk3b was downloaded from the PDB database. AutoDockTools 1.5.6 was used for the preparation of the crystal structure, including removing water molecules, separating the proteins, adding hydrogens, calculating the gasteiger charges, and saving this structure as a pdbqt format.

### 4.8. Molecular Docking

AutoDock vina software (version 1.1.2) was performed to verify the binding affinity of candidate targets to ROS by molecular docking simulation. The SDF format of ROS was downloaded from Pubchem. ROS was charged and energy-optimized using Chem 3D software (version 15.1), and the structures were saved in MOL 2 format. The pdbqt format of ROS was obtained by AutoDockTools 1.5.6. The binding energy was calculated, and the binding energy ≤ −5 kcal/mol was regarded as an effective combination. The conformation with the lowest binding energy was screened, and PyMOL 2.3 was used for visualization.

### 4.9. Statistical Analysis

The data are shown as the means ± SD for each group. For analysis of GI and defection, multi-group comparisons of mean values were performed using the one-way ANOVA with LSD, while the Kruskal–Wallis test was used when the variance was not uniform. SPSS 20.0 was used for data analysis. For 16S rDNA sequencing analysis, the Meta-stat 1.5 was used for statistical analysis of the significance of species differences between the two groups based on the Fisher exact test. A *p* < 0.05 is considered as statistical significance.

## 5. Conclusions

The administration of ROS effectively prevents constipation in mice by evaluating the gastro-intestinal transit rate, the number and weight of defecation events in six hours, and time to the first black stool defecation. The underlying mechanism may be through regulating the intestinal flora (the alterations of one specific family and six specific genera were found), reducing the serum levels of inhibitory neurotransmitters (VIP). Based on the network pharmacology, the targeted screening strategy further revealed 29 potential anti-constipation targets of ROS, and the network analysis showed Akt1, Stat3, Mapk8, Hsp90aa1, Cat, Alb, Icam1, Sod2, and Gsk3b can be regarded as the core targets in the ROS regulatory network, which mainly involved the inflammation, immunity, and antioxidation. In a word, ROS is a hopeful candidate for use as an anti-constipation prebiotic, and the combination of gut microbiota, enteric nervous system, and targeted screening strategy provide a novel perspective of ROS and the oligosaccharide to which it belongs.

## Figures and Tables

**Table 1:** PPI network topology analysis and corresponding binding energy.

Targets	Degree	Affinity (kcal/mol)
Akt1	10	−8.4
Stat3	7	−6.7
Mapk8	7	−7.5
Hsp90aa1	6	−6.4
Cat	5	−5.9
Alb	5	−6.6
Icam1	5	−5.5
Sod2	4	−5.5
Gsk3b	2	−6.6

## Data Availability

Data are contained within the article or supplementary material.

## Supplementary Materials

The following supporting information can be downloaded at: https://www.mdpi.com/article/10.3390/molecules27072235/s1, Figure S1: Ramachandran plot; Table S1: Intersection targets of ROS and constipation; Table S2: PPI network affected by the overlapped targets; Table S3: Biological process affected by the overlapped targets; Table S4: Cellular components affected by the overlapped targets; Table S5: Molecular function affected by the overlapped targets; Table S6: KEGG pathway affected by the overlapped targets; Table S7: Topological analysis of R-T-P network; Table S8: Homology modeling.

## Sample Availability

Samples of the compounds are not available from the authors.
